# Contributions of side effects to contraceptive discontinuation and method switch among Kenyan women: a prospective cohort study

**DOI:** 10.1111/1471-0528.17032

**Published:** 2022-01-18

**Authors:** CW Rothschild, BA Richardson, BL Guthrie, P Kithao, T Omurwa, J Mukabi, LS Callegari, EL Lokken, G John‐Stewart, JA Unger, J Kinuthia, AL Drake

**Affiliations:** ^1^ Department of Epidemiology University of Washington Seattle WA USA; ^2^ Departments of Biostatistics and Global Health University of Washington Seattle WA USA; ^3^ Division of Vaccine and Infectious Diseases Fred Hutchinson Cancer Research Center Seattle WA USA; ^4^ Departments of Global Health and Epidemiology University of Washington Seattle WA USA; ^5^ University of Washington‐Kenya Nairobi Kenya; ^6^ PATH‐Kenya Nairobi Kenya; ^7^ Departments of Obstetrics & Gynecology and Health Services University of Washington Seattle WA USA; ^8^ Department of Global Health University of Washington Seattle WA USA; ^9^ Departments of Global Health, Epidemiology, Medicine, and Pediatrics University of Washington Seattle WA USA; ^10^ Department of Obstetrics and Gynecology University of Washington Seattle WA USA; ^11^ Department of Research and Programs Kenyatta National Hospital Nairobi Kenya; ^12^ Present address: Population Services International Washington DC USA

**Keywords:** Contraception, contraceptive discontinuation, contraceptive switching, menstrual bleeding changes, sexual side effects, side effects

## Abstract

**Objective:**

To determine the contribution of specific contraceptive side effects to method switch and modern‐method discontinuation among Kenyan women.

**Design:**

A prospective cohort study.

**Setting:**

Five counties in Western Kenya.

**Participants:**

Women aged ≥18 years old and emancipated female minors ≥14 years old using modern, reversible contraception were recruited while attending 10 public health facilities.

**Methods:**

Patient‐reported adverse effect symptoms, method switch and discontinuation were reported through weekly text message‐based surveys for 24 weeks.

**Main outcome measurements:**

Prevalence, hazards ratio (HR).

**Results:**

Among 825 women, 44% were using implants, 43% injectables, 7% an intrauterine device and 6% oral contraceptive pills at enrolment. Most (61%) women were continuing a method used in the previous month. During the 24‐week follow up, incidence of contraceptive switch was 61.3 per 100 person‐years (95% confidence interval [CI] 52.4–71.8) and incidence of discontinuation was 38.5 per 100 person‐years (95% CI 31.6–47.0). On average, one‐quarter (prevalence [Pr] 0.24, 95% CI 0.22–0.26) of participants reported side effects or method problems weekly, with sexual side effects the most prevalent symptom (Pr 0.15, 95% CI 0.13–0.16). Lack of expected bleeding was associated with higher risk of method switch (adjusted hazard ratio [aHR] 2.36, 95% CI 1.22–4.57). Risk of all‐modern method discontinuation was higher among women experiencing irregular bleeding (aHR 2.39, 95% CI 1.20–4.77), weight changes (aHR 2.72, 95% CI 1.47–4.68) and sexual side effects (aHR 2.42, 95% CI 1.40–4.20).

**Conclusions:**

Addressing irregular bleeding, weight changes and sexual side effects through development of new products that minimise these specific side effects and anticipatory counseling may reduce method‐related discontinuation.

**Tweetable abstract:**

Bleeding, weight changes, sexual problems associated with discontinuation of #contraception, but many continue despite side effects.

## Tweetable abstract

Bleeding, weight changes, sexual problems associated with discontinuation of #contraception, but many continue despite side effects.

## Introduction

Understanding women’s contraceptive experiences is essential to develop and deliver family planning (FP) methods that match women’s needs and preferences. Side effects are a central aspect of women’s contraceptive decision‐making, contributing not only to physical discomfort and fear, but in some settings to significant sociocultural and economic hardships.^1^, ^2^, ^3^, ^4^, ^5^, ^6^ Despite recognition of the importance of side effects in contraceptive use, there are few longitudinal studies assessing side effects in low‐ and middle‐income countries (LMIC). Prospective data on the associations between specific contraceptive side effects and subsequent method switching or contraceptive discontinuation is critical for understanding the impacts of specific side effects and for developing contraceptive technology and programmes that are responsive to women’s preferences.

Worldwide, fear of side effects is the most common reason given for contraceptive non‐use among women who do not wish to become pregnant.^5^, ^7^, ^8^, ^9^ Less is known about determinants of method continuation, switch or discontinuation among women who experience side effects. Evidence suggests these associations may be context‐specific.^3^ At the country level, data from the Demographic and Health Surveys (DHS) reveal high between‐country variability in user‐reported discontinuation or non‐use due to side effects—from 9% in Mozambique to nearly 50% in Tanzania.^4^, ^7^, ^10^ Evidence from qualitative research emphasises that tolerability of side effects is influenced by a number of factors, including the severity and presentation of symptoms, individual preferences, desire for fertility control, as well as biological and contextual understanding of side effects.^11^, ^12^ For example, amenorrhoea may be viewed as an anticipated benefit of contraceptive use, or as an accumulation of ‘dirt’ in the body.^11^ Women with a strong desire to avoid pregnancy may be more likely to tolerate contraceptive side effects, particularly if they lack information or access to alternative methods.^13^ There is growing recognition of such trade‐offs between need for pregnancy prevention and contraceptive preferences, which may result in continued use despite method dissatisfaction.^11^, ^14^, ^15^

Use of modern contraceptive methods among women of reproductive age in Kenya has increased from 56% to 61% between 2019 and 2020 among married women and from 58% to 62% among unmarried, sexually active women over the same time period.^16^ Public FP services are generally free, with 93% of facilities delivering services without fees in a recent survey.^17^ Most contraceptive methods are readily available, with public service delivery outlets offering an average of seven different FP method types^17^; however, nearly half of public facilities experienced a stockout of at least one method on the date of the survey.^17^ Informal payments and provider bias have also been documented as barriers to women who wish to initiate, switch or discontinue contraceptive use.^18^, ^19^, ^20^ Furthermore, few studies have assessed Kenyan women’s preferences for, and responses to, specific contraceptive‐induced side effects.^6^ Understanding the contribution of side effects to contraceptive use behaviours in priority geographical regions such as Kenya is critical for honing global priorities for contraceptive method development and services.

We conducted a prospective cohort study among Kenyan women attending public FP or maternal child health (MCH) clinics who were using modern, reversible contraceptive methods to measure real‐time incidence of method switch and modern method discontinuation over 24 weeks and to assess associations between side effects and contraceptive switch or discontinuation.

## Methods

### Study population

Between February and May 2018, we enrolled women using modern, reversible contraception who were attending one of ten public FP or MCH clinics located in the Homa Bay, Kakamega, Kisumu, Nyamira, and Bungoma counties of Kenya. Adult women (≥18 years old) and emancipated female minors (≥14 years old with a prior pregnancy) were eligible to participate if they were currently using a modern, reversible contraceptive method,^21^ had daily access to a mobile phone with a Safaricom SIM card, and were able to read and respond to SMS messages in their preferred language (English, Swahili, Luo or Kisii) by themselves or with assistance. Women who were initiating, continuing or switching a modern contraceptive method were eligible. Study methods have been described previously.^22^ We restricted our analysis to participants using injectables, implants, intrauterine devices or systems (IUD/IUS) and daily oral contraceptive pills (OCP). Women with missing (or not adequately specified) baseline contraceptive methods or who were lost to follow up after enrolment were excluded from the analysis. We also excluded women who at enrolment stated a desire for pregnancy within 1 year.

### Data collection

Participants completed a short message system (SMS) survey administered at enrolment and weekly for 24 weeks. The enrolment survey captured sociodemographic characteristics and reproductive and contraceptive history, and follow‐up surveys collected data on weekly method use, reasons for method switch and discontinuation, and experiences of side effects. Participants received a single SMS reminder after 24 hours for incomplete surveys and were sent a 25 Kenyan Shilling (~ $0.25 USD) airtime credit upon completion of each survey.

### Ethical considerations and public involvement

The study was approved by the Ethical Review Committee of Maseno University, Kenya (MSU/DRPI/MUERC/00462/17). The study did not require approval from University of Washington’s (UW) Human Subjects Division (HSD) due to its ‘not engaged’ determination (STUDY00002934), but this analysis was approved by UW’s HSD (STUDY00008142). Participants provided written consent prior to study enrolment. Neither patients nor the public were involved in the development or design of the study. Local and national stakeholders from Kenya’s Ministry of Health and national reproductive health non‐governmental service provider organisations convened for a regional dissemination meeting of preliminary study findings held in March 2019.

### Ascertainment and definitions of key variables

#### Contraceptive side effects

Participants who reported experiencing any side effects or problems with their current contraceptive method in the past week (or were unsure) were asked a series of method‐specific questions about symptoms. We measured the following specific side effects: heavy or prolonged bleeding, irregular bleeding, lack of expected bleeding, abdominal or back pain (including painful menses and cramping), changes in weight (either gain or loss) and sexual side effects, including problems related to sexual pleasure, libido or discomfort during intercourse. Participants were asked to report if specific side effects occurred in the past week, with the exception of weight changes, which was asked about in the past month. To standardise capture of the experience of side effects across contraceptive use outcomes (continuation, switch and discontinuation), side effects in each study week except the first were defined based on the most recent available survey conducted in the prior 4 weeks. In the first week of follow up, exposure to side effects was captured using the week 1 survey for women continuing or discontinuing their method (who were asked to report on experiences in the past week); side effects for women who switched methods in the first week of follow up are missing, as experience of side effects was not ascertained for the method used at enrolment. Side effects were coded as missing if all surveys in this interval were incomplete or ‘not sure’/refused responses. Additional details are provided in Appendix S1 and Table S1.

#### Discontinuation and method switch

We defined all‐modern method discontinuation as a period of at least two consecutive weeks when the participant reported not using any modern contraceptive method. The 2‐week period required for discontinuation was selected to minimise outcome misclassification based on single‐week entry errors and to allow single weeks of method interruption (such as during method resupply), while maintaining sensitivity to detect short intervals of non‐use. Method switch was defined as using a different modern method type than reported at enrolment, where modern methods were defined to include injectables, implants, IUD/IUS, OCP, the emergency contraceptive pill, condoms (male and female), diaphragm, sterilisation, lactational amenorrhoea, Standard Days Method and the Two‐Day Method.^21^ We required reported use of the new modern method for a period of at least four consecutive weeks in order to minimise misclassification of method switching due to entry errors (Appendix S1).

#### Other covariates of interest

Strength of desire to avoid pregnancy was defined based on a single question captured at enrolment that asked how much of a problem it would be if they were to become pregnant in the next few weeks (big, small or no problem). Participants were considered contraceptive naïve if they reported no prior FP use at enrolment. Method satisfaction was defined based on a 5‐point Likert scale of overall satisfaction with the current method (very dissatisfied, dissatisfied, neutral, satisfied or very satisfied).

### Statistical analysis

We estimated incidence and adjusted hazard ratios (aHR) of method switch and discontinuation by each side effect using cause‐specific Cox proportional hazard models.^23^ Adjusted models included *a priori* potential confounders (marital status, contraceptive method at enrolment, age, FP user type [initiator, switcher or continuer at enrolment] and postpartum status [end of last pregnancy <6 months ago]) and were stratified by enrolment facility.^24^ We fit cause‐specific Cox proportional hazards models, which treat method switch as a censoring event in discontinuation models, and discontinuation as a censoring event in switch models. We assessed robustness of the cause‐specific modelling approach in sensitivity analyses using Fine–Gray competing risks models.^25^

We combined last observation carried forward with next observation carried backwards to fill to the mid‐point of the missing intervals of contraceptive use or method type, but did not impute monotonic right censoring^26^; imputed data was used to define discontinuation and switch. We used a complete case approach to missing side effects and baseline covariates in primary models but conducted a sensitivity analysis using multiple imputation with chained equations (MICE)^27^ to impute missing side effects. In addition, we conducted a sensitivity analysis in which all missing observations of side effects were coded as not experienced.

We calculated attributable risk and population attributable risk percentages with 95% confidence intervals (CI). We estimated mean weekly prevalence of side effects by contraceptive use outcome (continuation, switch or discontinuation) using log‐binomial generalised estimating equations with an independent correlation structure, robust standard errors and indicator variables for week of follow up and contraceptive use outcome.

In exploratory analyses, we assessed effect modification of the association between side effects, and discontinuation and switch by the following potential effect modifiers: youth, gravidity, contraceptive naivety and strength of desire to avoid pregnancy. We also determined the association between method dissatisfaction and method switch, and discontinuation, using cause‐specific Cox models analogous to the primary side effects models. We conceptualised side effects as a confounder of the relationship between dissatisfaction and switch (and discontinuation), so additionally adjusted for experience of any side effects.

## Results

Of 1212 women enrolled, we included 825 (68%) in our analysis (Figure S1). The most common reasons for exclusion were completely missing follow up (14%, *n *= 171) or a missing/refused response to fertility intentions at enrolment (11%, *n *= 138). Approximately half (48%) of participants were between the age of 25–34 years, with 39% <25 years (Table 1). Most (82%) were married and had at least one prior pregnancy (94%), with 29% reporting the end of their most recent pregnancy within 6 months of enrolment. Nearly half (47%) reported breastfeeding at baseline. At enrolment, implants (44%) and injectables (43%) were the most common methods used. The majority (61%) of women were continuing method users. Half (52%) of women reported having ever experienced contraceptive side effects in their lifetime. The majority (56%) reported that becoming pregnant in the next few weeks would be a ‘big problem’. Over 70% (487/684) of participants reported experiencing side effects or method problems at least once during follow up; experience of specific side effects over follow up ranged from 35% for heavy/prolonged bleeding to 51% for sexual side effects (Table S2). Experience of sexual side effects during follow up ranged from 41% among OCP users to 53% among implant users. Across method types, 41–47% of participants reported experiencing problems with libido or sexual pleasure at least once during follow up (Table S3). Painful intercourse was reported by 40–45% of implant, Cu‐IUD and injectable users but only 21% of OCP users.

**Table 1:** Characteristics of female contraceptive users at study enrolment

	Total *n*	Overall
		*n* (%) or median (IQR)
Age (years)	810	26 (23, 31)
Age category		
<25	810	313 (39)
25–34		391 (48)
>34		106 (13)
Married (legal or presumed)	822	671 (82)
Completed education (years)	823	8 (4, 13)
Nulligravid	817	50 (6)
<6 months since most recent pregnancy	817	237 (29)
Currently breastfeeding	809	381 (47)
Method type		
Implant	825	360 (44)
Cu‐IUD		60 (7)
Injectable		354 (43)
OCP		51 (6)
FP user type		
Initiating contraception	819	212 (26)
Switching method		105 (13)
Continuing method used in past month		502 (61)
Ever experienced contraceptive side effects or method problems*		
No	756	241 (32)
Yes		392 (52)
Contraceptive naïve		123 (16)
Fertility intentions		
Wants no children in the future	825	184 (22)
Not sure if wants children in the future		68 (8)
Yes, not sure of preferred timing of next pregnancy		100 (12)
Yes, next pregnancy in 1–2 years		77 (9)
Yes, next pregnancy in >2 years		396 (48)
Becoming pregnant in the next few weeks would be a		
Big problem	815	453 (56)
Small problem		67 (8)
No problem		186 (23)
Not sure		109 (13)
Received all three components of Method Information Index (MII)** (ref: <3)	794	455 (57)

Cu‐IUD, copper intrauterine device; IQR, interquartile range; OCP, oral contraceptive pills.

The analysis is restricted to women who did not report desiring their next pregnancy within 1 year and who were using implants, injectables, IUD or OCP at baseline. For the 59/818 (7%) of participants who reported using a second method of family planning, their primary method was used to define method switch in this analysis. Of participants using a dual method at study enrolment, 33/53 (63%) were using condoms, 17/53 (32%) fertility awareness‐based methods, and 3/53 (6%) other methods.

* At enrolment, women were asked whether they had ever used family planning before the current day in their lifetime. Women were then asked about use of specific modern, reversible methods (injectables, implants, IUD, OCP and condoms). For each method for which they reported lifetime use, they were asked about experience of any side effects or problems with the method. History of contraceptive side effects is coded as ‘yes’ if women reporting experiencing side effects with any of the individual methods. FP naïvety was defined as reporting no history of FP use prior to the date of enrolment and initiating a method on that day. Women reporting no prior FP use but continuing/switching a method on the date on enrolment were coded as missing, as they were not administered questions on method‐specific side effects history.

** The MII is coded as a binary variable equal to 1 if women reported received all three of the following counselling components during the FP visit on the date of study enrolment (or at her most recent visit, if she did not receive FP services on the date of enrolment): information on other methods, side effects of selected method, and what to do if side effects occur; the variable is coded as 0 if <3 counseling items were reported.

### Method switching

**Figure 1:**
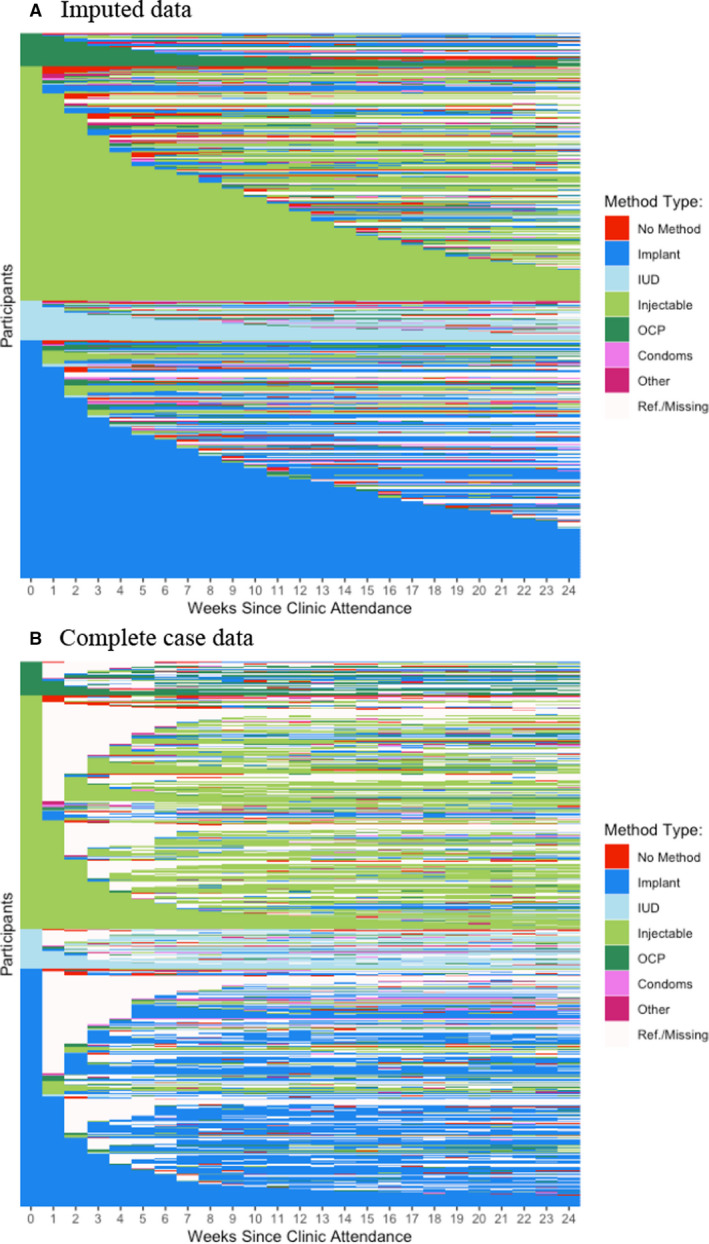
Patterns of contraceptive method use over 24 weeks. (A) Imputed data. (B) Complete case data.

We observed 254.3 woman‐years of follow up and 156 method switches, for an overall incidence rate of 61.3 per 100 woman‐years (95% CI 52.4–71.8) (Figure S2, Table S4). Descriptive patterns of contraceptive method use are presented in Figure 1. Switches occurred equally from LARC to non‐LARC methods (40%) and vice versa (38%). We did not detect differences in the unadjusted risk of switch between women using Cu‐IUDs (hazard ratio [HR] 1.13, 95% CI 0.60–2.12) or injectables (HR 1.18, 95% CI 0.80–1.75) compared with women using implants; however, risk of switch was two‐fold higher among OCP than among implant users (HR 2.12, 95% CI 1.18–3.81) (Tables S5 and S6). Among women who self‐reported switching methods and provided a reason for doing so (49%, 77/156), the most frequently reported reason was side effects (61%, 47/77), followed by desire for a more effective method (19%, 15/77).

**Figure 2:**
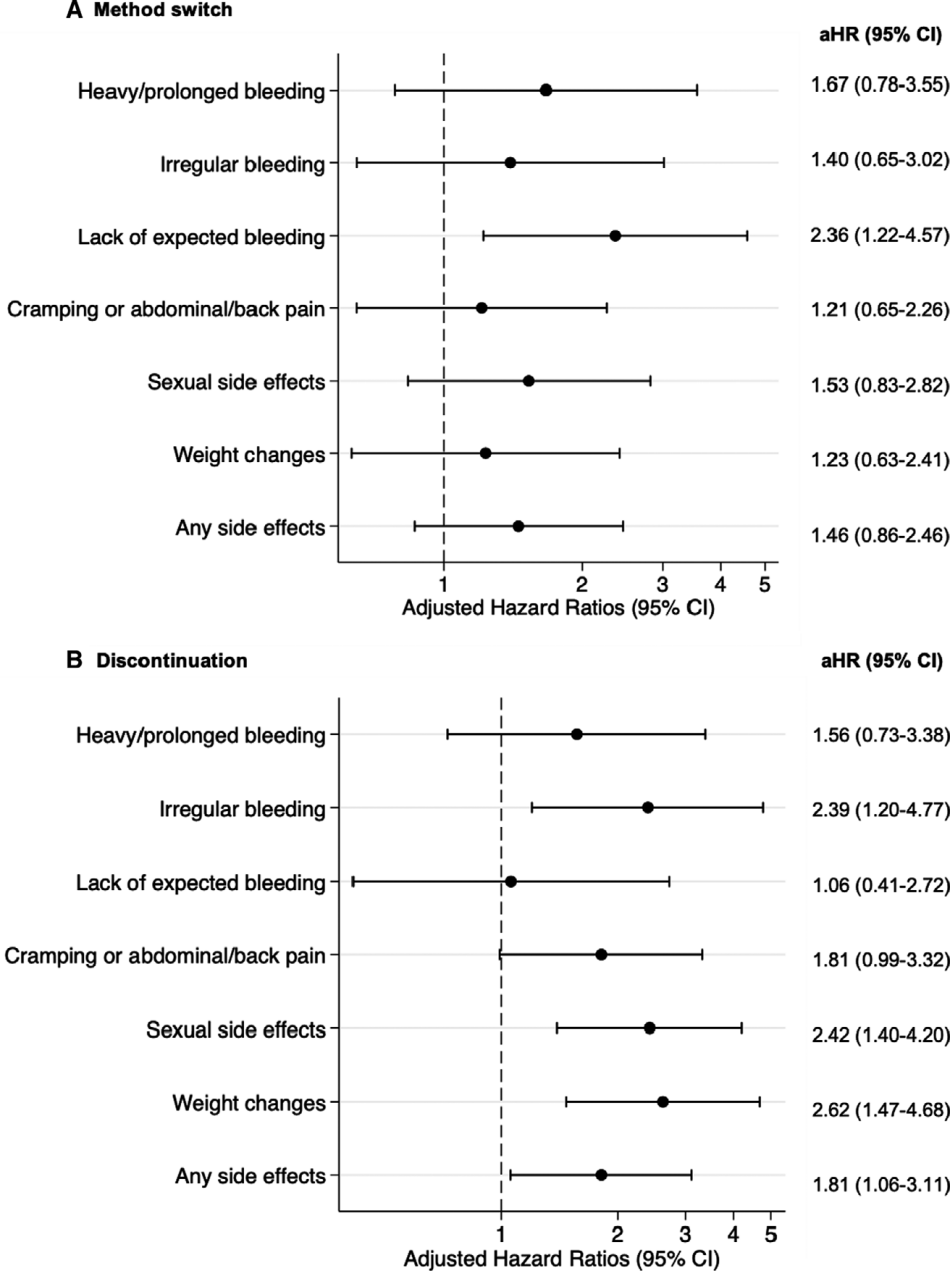
Adjusted hazard ratios of method switch and discontinuation by side effects experience. Cause‐specific hazard ratios estimated using Cox proportional hazards models stratified by enrolment facility. All models are adjusted for the following baseline covariates: marital status, contraceptive method type, age (in years), years of completed education, FP user type (initiator, continuer, switcher at baseline) and postpartum status (end of pregnancy within 6 months of study enrolment). Adjusted models comprise 636–642 participants, 63–65 switch events and 56–59 discontinuation events due to missing values in the side effects exposure and covariates.

Point estimates of weekly prevalence of side effects were higher among women who eventually switched methods than those who continued using their initial method (Table 2). However, only lack of expected bleeding was significantly associated with switch in adjusted analyses (aHR 2.36, 95% CI 1.22–4.57) (Figure 2). Sensitivity analyses produced similar results (Figures [Link], [Link], [Link]).

**Table 2:** Weekly probability of side effects, by contraceptive use outcome

	Continued	Switched	Discontinued	Full sample
	Prevalence (95% CI)	Prevalence (95% CI)	Prevalence (95% CI)	Prevalence (95% CI)
Heavy/prolonged bleeding	0.07 (0.06–0.08)	0.11 (0.07–0.18)	0.11 (0.07–0.18)	0.07 (0.06–0.09)
Irregular bleeding	0.08 (0.06–0.09)	0.14 (0.09–0.22)	0.16 (0.10–0.24)	0.08 (0.07–0.09)
Lack of expected bleeding	0.07 (0.06–0.08)	0.12 (0.08–0.19)	0.07 (0.04–0.13)	0.07 (0.06–0.08)
Cramping or abdominal/back pain	0.11 (0.10–0.13)	0.24 (0.17–0.34)	0.20 (0.14–0.30)	0.12 (0.11–0.14)
Sexual side effects	0.14 (0.12–0.16)	0.26 (0.18–0.36)	0.22 (0.15–0.32)	0.15 (0.13–0.16)
Weight changes	0.12 (0.11–0.14)	0.19 (0.12–0.28)	0.20 (0.13–0.30)	0.13 (0.11–0.15)
Any side effects or method problems	0.23 (0.21–0.25)	0.36 (0.28–0.48)	0.35 (0.26–0.47)	0.24 (0.22–0.26)

(A) Participants are defined based on their contraceptive use outcome (method switch, discontinuation or censoring, whichever occurred first). Side effects are defined using the participant’s weekly report. Reported side effects for the week of method switch are excluded (as these capture side effects of the new method). Mean weekly prevalence and corresponding 95% confidence intervals were estimated using log‐binomial generalised estimating equations adjusted for contraceptive use outcome (continued, switched, discontinued). Note that participants were censored due to switch, discontinuation, non‐response or loss to follow up. Analyses comprise 711–718 participants due to missing values in side effects.

We detected evidence of age as an effect modifier of the associations between sexual problems, heavy bleeding and any side effects and risk of method switch. Among young women <25 years old, these specific side effects were not significantly associated with method switch (sexual side effects: aHR 0.41, 95% CI 0.11–1.56; heavy bleeding: aHR 0.35, 95% CI 0.05–2.58; any side effects: aHR 0.61, 95% CI 0.23–1.59) (Table S7, Figure S6). However, among older women (age ≥25), side effects were associated with 1.7‐ to 3.5‐fold risk of switch (sexual side effects: aHR 3.52, 95% CI 1.78‐4.07; heavy bleeding: aHR 3.41, 95% CI 1.52–7.66; any side effects: aHR 2.71, 95% CI 1.30–4.41). We detected evidence of similar effect modification by gravidity (for all specific side effects except cramping and weight changes) and first‐time contraceptive use (for irregular bleeding), although the precision of our estimates of these interactions was limited by the small number of nulligravid participants (*n *= 50, 6%) (results not shown). We did not find evidence of effect modification by strength of desire to avoid pregnancy or statistically significant associations between method dissatisfaction and switch (Table S8).

### Discontinuation

There were 98 discontinuation events, for an overall incidence of 38.5 per 100 woman‐years (95% CI 31.6–47.0). Among 38 (39%) women who discontinued and provided a reason, desire for pregnancy (34%) was the most frequently cited reason, despite excluding women who stated at enrolment that they were not planning to become pregnant in the next year. Side effects (24%) was the next most common reason. Risk of discontinuation was 2.5‐fold higher among OCP (HR 2.46, 95% CI 1.30–4.65) than among implant users. We did not detect differences in risk of discontinuation between Cu‐IUD or injectable users compared with implant users.

We detected higher risk of discontinuation among women with irregular bleeding (aHR 2.39, 95% CI 1.20–4.77), weight changes (aHR 2.72, 95% CI 11.47–4.68) and sexual side effects (aHR 2.42, 95% CI 1.40–4.20) compared with women who did not report these side effects. Results were robust in sensitivity analyses using multiple imputation and Fine–Grey competing risks models.

Nearly three‐fourths (71%) of women reported experiencing side effects or method problems at least once during follow up. Sexual problems were the most prevalent side effects reported (Pr 0.15, 95% CI 0.13–0.16), followed by weight changes (Pr 0.13, 95% CI 0.11–0.15), cramping or abdominal/back pain (Pr 0.12, 95% CI 0.11–0.14) and irregular bleeding (Pr 0.08, 95% CI 0.07–0.09). In this population, eliminating all contraceptive side effects experienced in the past month would be expected to reduce overall discontinuation by 18% (95% CI 5–29%) (Table S8). Sexual problems (population attributable fraction [PAF] 17%, 95% CI 10–23%) and weight changes (PAF 18%, 95% CI 11–24%) make the largest contributions to discontinuation and explain approximately half (46–59%) of discontinuation among women who experience these side effects.

Among women with a strong desire to avoid pregnancy (reported that getting pregnant in the near future would be a ‘big problem’), side effects were not associated with discontinuation. Among women with a weak desire to avoid pregnancy (getting pregnant would be a ‘small problem’ or ‘no problem’), experience of side effects was associated with 2.8‐ to 4.8‐fold risks of discontinuation (aHR 2.81, 95% CI 1.06–7.48 for weight changes; aHR 3.82, 95% CI 1.59–9.17 for sexual problems; aHR 3.78, 95% CI 1.60–8.95 for cramping or abdominal pain; aHR 4.83, 95% CI 1.78–13.05 for irregular bleeding). However, effect modification was not statistically significant at the α = 0.05 level. We found evidence of statistically significant effect modification of the association between lack of expected bleeding and discontinuation by both nulligravida and first‐time contraceptive users, although estimates were imprecise. The association between side effects and discontinuation was similar in the various age groups. Relative to women who were ‘very satisfied’ with their method, we found that being ‘very dissatisfied’ was associated with increased risk of discontinuation (aHR 4.54, 95% CI 1.30–15.88).

## Discussion

Contraceptive side effects, method switch and discontinuation were common in a cohort of women using modern, reversible contraception in Western Kenya who did not want to become pregnant within the next year. Method switching has often been estimated in combination with all‐method discontinuation, or defined to allow long gaps in method use that may correspond to substantial risk of unintended pregnancy. Here, we leveraged novel prospective data collected weekly to highlight modern method switching occurring within short time intervals, an outcome which can be viewed as a positive indication of women’s knowledge of and ability to tailor methods to preferences.^28^ By this definition, we find that one‐quarter (25%) of women switched contraceptive methods by 24 weeks after FP/MCH clinic attendance. An additional 16% discontinued all modern contraception during this period. Rates of switch and discontinuation were lowest among implant users and highest among OCP users, consistent with other studies.^2^, ^4^, ^29^, ^30^, ^31^ In other studies from Kenya, method discontinuation was ~30%, 6–12 months after contraceptive initiation.^32^, ^33^ In the multi‐country HIV‐1 prevention ASPIRE trial, cumulative 1‐year discontinuation was 38% among DMPA users and 48% among OCP users,^29^ comparable to the 24‐week cumulative incidence in our study of 17% and 36%, respectively. Implant users in our study reported higher rates of method switching (21%) and discontinuation (12%) than the combined rate of 11% for method switch and discontinuation in ASPIRE by 6 months. ASPIRE participants received ongoing contraceptive counselling to promote continuation, which may explain lower rates of switch and discontinuation.

Of all contraceptive side effects, changes in menstrual bleeding have received the most attention by researchers and healthcare providers.^6^ Although bleeding side effects have been associated with discontinuation,^6^, ^34^ several studies among IUD and OCP users have found that bleeding side effects did not influence discontinuation.^35^, ^36^ We found irregular bleeding increased risk of discontinuation, but not method switch. Conversely, lack of expected bleeding was associated with switch, but not discontinuation. This is consistent with prior evidence from Kenya of high method continuation rates among injectable users experiencing amenorrhoea.^37^ Relatively low discontinuation among amenorrhoeic women may reflect receipt of anticipatory side effects counselling, which has been shown to significantly reduce discontinuation rates in other settings.^38^ Sexual side effects and weight changes were each associated with discontinuation and accounted for a higher proportion of overall discontinuation compared with irregular bleeding. Contraceptive research has paid relatively little attention to the impacts of contraception on women’s sexual pleasure and experiences, particularly in LMICs.^39^, ^40^, ^41^, ^42^ We found that among women using implants, Cu‐IUD, injectables and OCP, sexual side effects were reported at least once over follow up by between 41% and 53% of women using each method. These findings are consistent with prior reports of sexual dysfunction being common and not varying by method type among implant, Cu‐IUD and injectable users.^43^ Our findings build upon previous findings that highlight the importance of sexual experience in contraceptive acceptability and sustained use, and the need both to prioritise sexual pleasure and functioning in new contraceptive technology development and to improve counselling along the care continuum for affected women.^41^, ^42^

The prevalence of side effects reported in our study population was high, with nearly one‐quarter of women experiencing at least one side effect or method‐related problem weekly. Despite the high burden of side effects, many women continued using their initial contraceptive method. Our findings are consistent with recent evidence from a cohort study in India, which found that half of women who experienced side effects were still using their initial method at 6 months.^44^ Adjusting for side effects, risk of discontinuation was higher among women who felt ‘very dissatisfied’ with their method but not among those who were less than fully satisfied with their method. Our findings suggest that women’s needs for pregnancy prevention often outweigh the physical, psychological and other costs of contraceptive use. There are several explanations for continued method use among women experiencing side effects: supply factors such as limited range of available methods, time and monetary costs, and provider coercion may all limit women’s ability to switch or discontinue methods.^14^, ^45^, ^46^ On the demand side, continuation may be driven by perceptions of side effects as tolerable, inconsequential or even beneficial,^6^ gaps in contraceptive knowledge, specific contraceptive needs (such as concealability), perceived risk of pregnancy with method switch or previous poor experiences with other methods.

Notably, we found stark differences in women’s responses to side effects based on the strength of their desire to avoid pregnancy, even among those who expressed no short‐term intention to become pregnant. In exploratory analyses, we found nearly two‐ to five‐fold risk of discontinuation associated with side effects among women who expressed a weak desire to avoid pregnancy, but no association among women with a strong desire to avoid pregnancy. These findings highlight the shortcomings of conceptualising continued use as method acceptability, and the need for continued outreach to address contraceptive users’ dynamic needs and experiences over time.

Individual characteristics may also prevent women from effectively switching methods despite experiencing side effects. We found that method switch was more common among older women who experienced any side effects, sexual side effects and heavy bleeding, but not among young women. While these exploratory analyses should be interpreted with caution, our findings suggest that young women experiencing side effects may face unique barriers to timely and effective method switching. Young women may also perceive specific side effects to be less ‘bothersome’ than do older women. Future research is needed to elucidate further the main factors, including those at the health systems level, driving continued method use among women experiencing side effects.

Our study has several strengths. We utilised a unique, prospective design with high‐frequency capture of side effects among all contraceptive users, providing insight into a complex phenomenon. The cohort’s method use closely resembles the national modern reversible contraceptive method mix.^16^ The prospective design and weekly surveying tempo gave us unique ability to detect dynamic experiences of contraceptive use. We were able to explore heterogeneity in the associations between side effects, switch and discontinuation by specific types of side effects. While much of the published literature defines discontinuation at the month level,^4^, ^33^ weekly surveys permitted focus on definitions of method switching and discontinuation that identify short‐term gaps in method use that may represent important pregnancy risks in a cohort of women who wish to delay pregnancy for at least 1 year.

Our study is also subject to limitations. Method misclassification is possible due to entry errors using the SMS survey for data collection and inability to correct for inconsistent responses.^47^ However, we expect misclassification to be non‐differential, biasing findings towards the null. This may have limited our ability to detect true associations. In addition, we did not capture side effect experience among women who switched in the first week of follow up. While results from primary models were robust to multiple imputation, imputation models retained missing values due to the presence of participants with completely missing side effects data over follow up: ascertainment of exposure to side effects was missing for one‐third and nearly half of weekly observations in which discontinuation and switch occurred, respectively. Our study is also limited by the short duration of follow up (24 w) and limited number of IUD and OCP users. Failure to ascertain changes in fertility preferences, partner status and sexual activity over time may also have resulted in residual confounding. We did not capture changes in breastfeeding behaviours over follow up; it is plausible that breastfeeding cessation or changes to contraceptive eligibility among postpartum women may have influenced method switch. Finally, reliance on brief but high‐frequency surveys prevented capture of perceptions of the severity of side effects and the degree to which women perceived side effects as bothersome. We also did not measure the individual’s ability to access affordable contraceptive services should they wish to switch or discontinue their current method (which, in the case of LARC methods, requires provider removal). All of these may be important explanatory factors in switch and discontinuation.^44^, ^48^ Even where contraceptive services are available and generally free, recent evidence from Kenya has shown that provider barriers to LARC removal may restrict women’s ability freely to decide to switch or stop LARC use.^18^

## Conclusion

Contraceptive acceptability has previously been conceptualised as sustained use of a contraceptive method.^13^ However, this definition fails to recognise that women continue using methods that align poorly with their contraceptive preferences. Our findings suggest that specific contraceptive user groups, including women who are young and have a strong desire to avoid pregnancy, often continue using methods despite experiencing side effects. As a result, FP providers and policymakers should not equate contraceptive continuation with method acceptability or satisfaction when tracking programme successes. Global FP initiatives must re‐centre their objectives to focus on meeting women’s contraceptive preferences in addition to their needs for pregnancy prevention. This requires a critical examination of how current metrics, programmes and policies may fail to identify contraceptive dissatisfaction and barriers to method switch and discontinuation among women who experience method‐related challenges.

### Disclosure of interests

CWR was engaged as a subject matter expert consultant with the Bill and Melinda Gates Foundation. The work performed was unrelated to the content of this manuscript. Completed disclosure of interests form available to view online as supporting information.

### Contribution to authorship

CWR and ALD secured funding for the study and were involved in study design, coordinated data collection and validation of the data, participated in the analysis, and contributed to writing the article. PK coordinated data collection. JUG contributed to designing the study, validation of the data, data analysis and writing the article. BAR, BLG and ELL participated in the analysis and contributed to writing the article. JM and TO contributed to study design and data collection. GJS and JK contributed to study design and to writing the article. All authors reviewed and approved the final manuscript.

### Funding

Support for this research came from the Eunice Kennedy Shriver National Institute of Child Health & Human Development of the National Institutes of Health (F31HD097841 [CR] and a research infrastructure grant, P2C HD042828, and a training grant, T32 HD101442‐01, to the Center for Studies in Demography & Ecology at the University of Washington), the National Institute of Allergy and Infectious Diseases of the National Institutes of Health (K01AI11628 [ALD], F32HD100202 [EML]). The content is solely the responsibility of the authors and does not necessarily represent the official views of the National Institutes of Health or of the Bill & Melinda Gates Foundation.

### Acknowledgements

The authors would like to thank the mCUBE Study research participants and study team for their invaluable contributions. We would also like to acknowledge the support of the Global Center for Integrated Health of Women, Adolescents, and Children (Global WACh) in the University of Washington Department of Global Health and the Center for Studies in Demography and Ecology (CSDE) at the University of Washington. We would like to give special thanks to Elizabeth Harrington, MPH MD, for her advice on definitions and measurement of contraceptive side effects and method

## Supporting information


**Figure S1.** Study flow.

**Figure S2.** Kaplan–Meier estimates of discontinuation and method switch, by method type used at enrolment.

**Figure S3.** Sensitivity analysis: Fine–Grey competing risk survival models.

**Figure S4.** Sensitivity analysis: cause‐specific hazard models using multiple imputation to address missing adverse effects exposure.

**Figure S5.** Sensitivity analysis: all missing adverse effects exposure values set to ‘unexposed’.

**Figure S6.** Effect modification of adverse effects.

**Table S1.** Adverse effects symptom ascertainment.

**Table S2.** Percent of weeks prior to method switch or discontinuation in which participants reported adverse effects.

**Table S3.** Percent of weeks prior to method switch or discontinuation in which participants reported sexual adverse effects, by symptom type.

**Table S4.** Contraceptive use dynamics over 24 weeks.

**Table S5.** Discontinuation and method switch by baseline characteristics.

**Table S6.** Incidence of modern‐method discontinuation and switch.

**Table S7.** Effect modification of adverse effects on switch and discontinuation.

**Table S8.** Associations between level method satisfaction and switch and discontinuation.

**Appendix S1.** Details of study design, procedures and variable ascertainment.

Supplementary Material

Supplementary Material

Supplementary Material

Supplementary Material

Supplementary Material

Supplementary Material

Supplementary Material

Supplementary Material

Supplementary Material

Supplementary Material

Supplementary Material

## Data Availability

Data are available upon request at Rothschild, Claire; Drake, Alison; mCUBE Study Team, 2021, “Mobile Data Collection of Contraceptive Uses, Behaviors, and Experiences in Western Kenya (mCUBE) Study”, https://doi.org/10.7910/DVN/N8U13U, Harvard Dataverse, DRAFT VERSION, UNF:6:AQszXTtNi5BnhHPs4it8rQ== [fileUNF]
