# Supplementary figures and images for: Contributions of side effects to contraceptive discontinuation and method switch among Kenyan women: a prospective cohort study
Source: BJOG. 2022 Jan 18;129(6):926–37. doi: 10.1111/1471-0528.17032 (PMC9035040; doi:10.1111/1471-0528.17032)

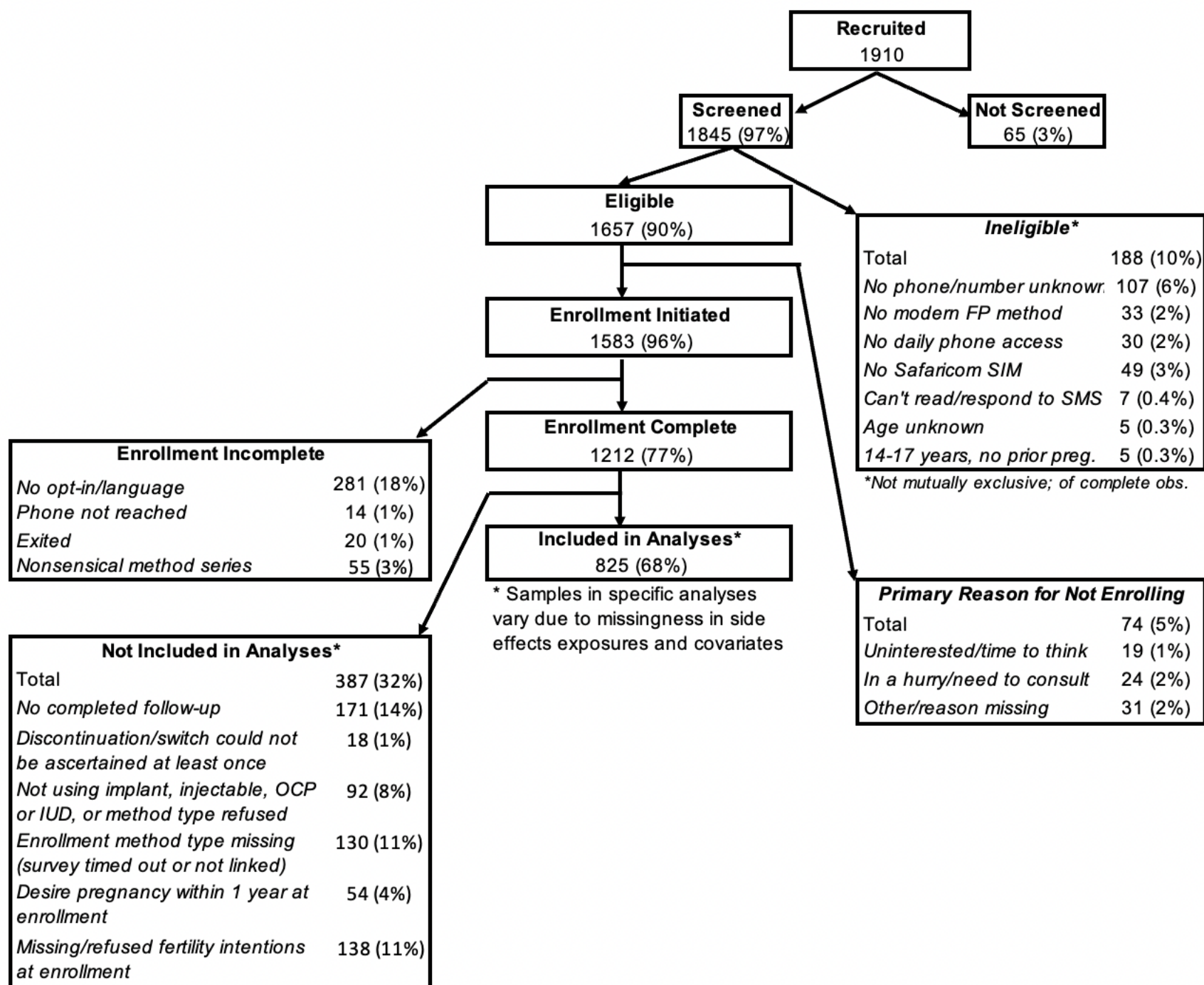

\* Not mutually exclusive

Supplement: Supplementary file 1 — Figure S1. Study flow. [file BJO-129-926-s020.pdf]
