# Supplementary material for: Contributions of side effects to contraceptive discontinuation and method switch among Kenyan women: a prospective cohort study
Source: BJOG. 2022 Jan 18;129(6):926–37. doi: 10.1111/1471-0528.17032 (PMC9035040; doi:10.1111/1471-0528.17032)
Supplement: Supplementary file 2 — Figure S2. Kaplan–Meier estimates of discontinuation and method switch, by method type used at enrolment. [file BJO-129-926-s013.docx]

**S2 Fig. Kaplan-Meier estimates of discontinuation and method switch, by method type used at enrollment**

**Panel A. Method switch**


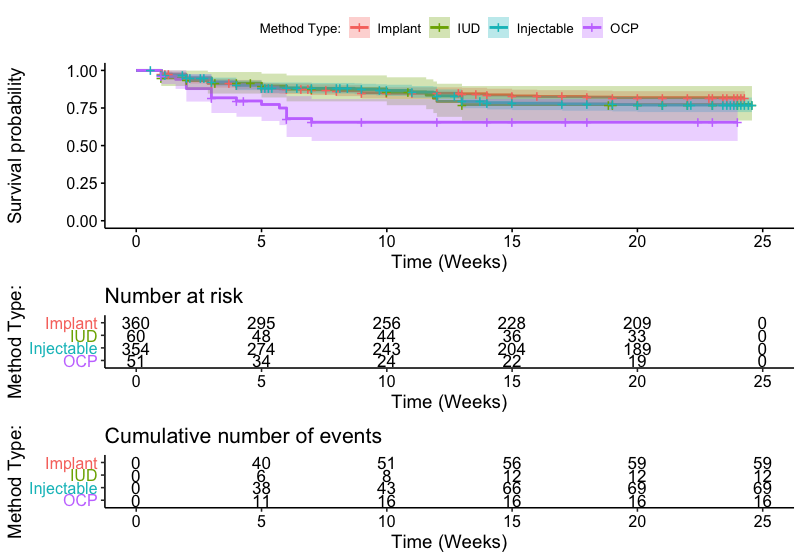


**Panel B. Discontinuation**


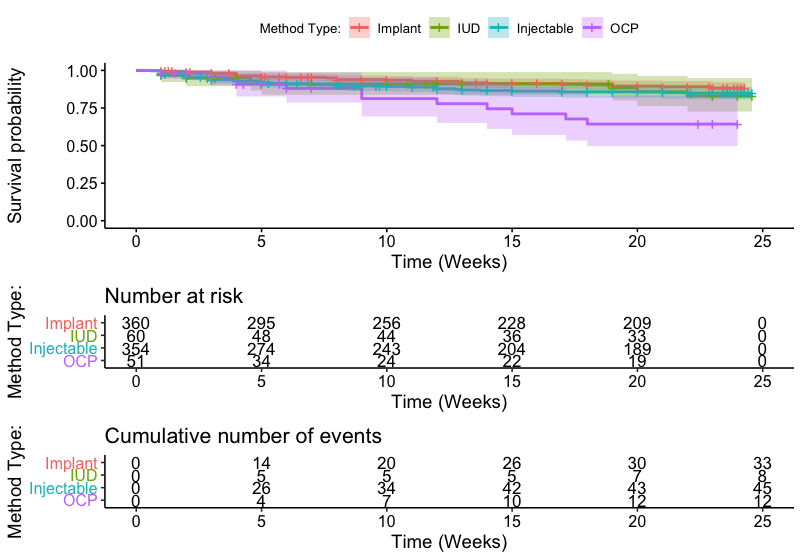


Notes: Kaplan-Meier estimates of survival probability by method type used at enrollment. Number of participants at risk over time is identical for the switch (Panel A) and discontinuation (Panel B) outcomes, as individuals are considered censored in the case of a competing event. Shaded areas represent 95% confidence intervals.
