# Supplementary material for: Contributions of side effects to contraceptive discontinuation and method switch among Kenyan women: a prospective cohort study
Source: BJOG. 2022 Jan 18;129(6):926–37. doi: 10.1111/1471-0528.17032 (PMC9035040; doi:10.1111/1471-0528.17032)
Supplement: Supplementary file 3 — Figure S3. Sensitivity analysis: Fine–Grey competing risk survival models. [file BJO-129-926-s015.docx]

**S3 Fig: Sensitivity Analysis: Fine-Grey competing risk survival models**

Panel A. Adjusted subdistribution hazards ratios of method switch

Panel B. Adjusted subdistribution hazards ratios of discontinuation

Notes: Adjusted subhazard ratios are estimated using Fine-Grey proportional hazards models for competing risks, with switch defined as the competing risk for discontinuation models and vice versa. Side effects are those reported in the past week for women discontinuing or continuing a method, and in the week prior to the past week (for women switching a method). All models are adjusted for the following covariates assessed at study enrollment: marital status, contraceptive method type, age (in years), years of completed education, FP user type (initiator, continuer, switcher at baseline), and postpartum status (end of pregnancy within 6 months of study enrollment). Adjusted models comprise 636-642 participants due to missing values in the time-varying side effects exposure and baseline covariates.
