# Supplementary material for: Contributions of side effects to contraceptive discontinuation and method switch among Kenyan women: a prospective cohort study
Source: BJOG. 2022 Jan 18;129(6):926–37. doi: 10.1111/1471-0528.17032 (PMC9035040; doi:10.1111/1471-0528.17032)
Supplement: Supplementary file 4 — Figure S4. Sensitivity analysis: cause‐specific hazard models using multiple imputation to address missing adverse effects exposure. [file BJO-129-926-s023.docx]

**S4 Fig: Sensitivity Analysis: Cause-specific hazard models using multiple imputation to address missing side effects exposure**


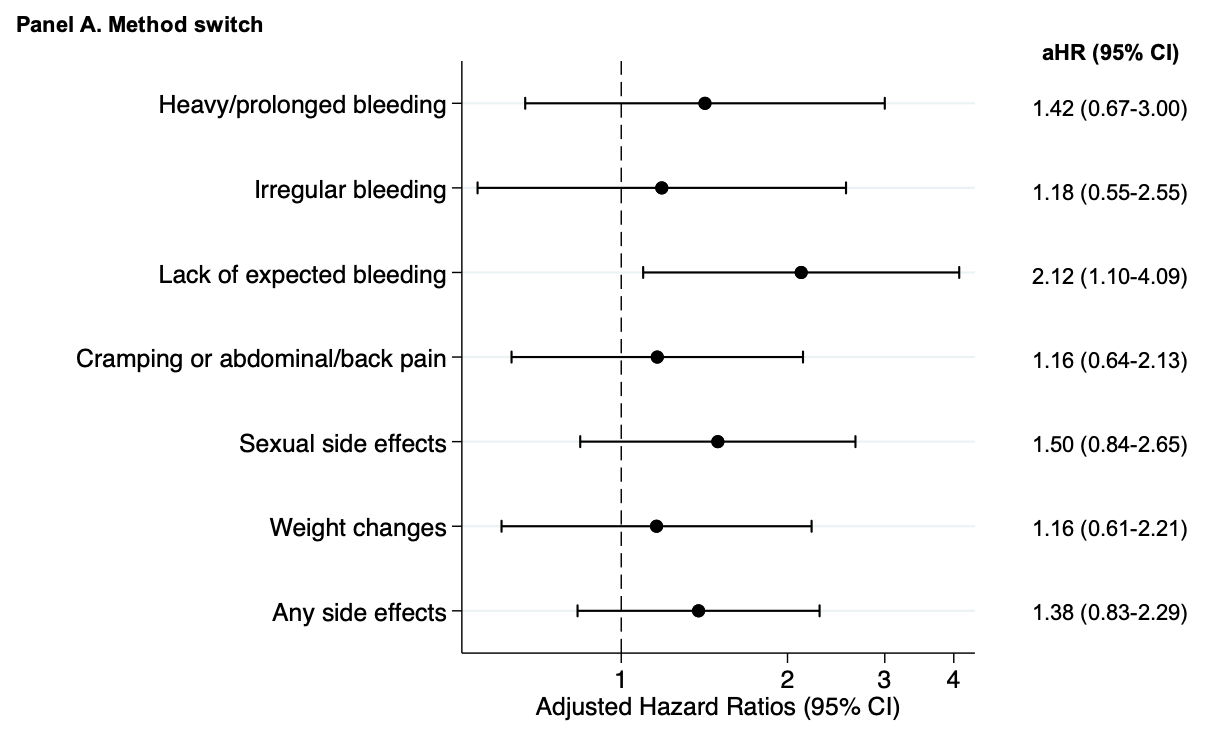


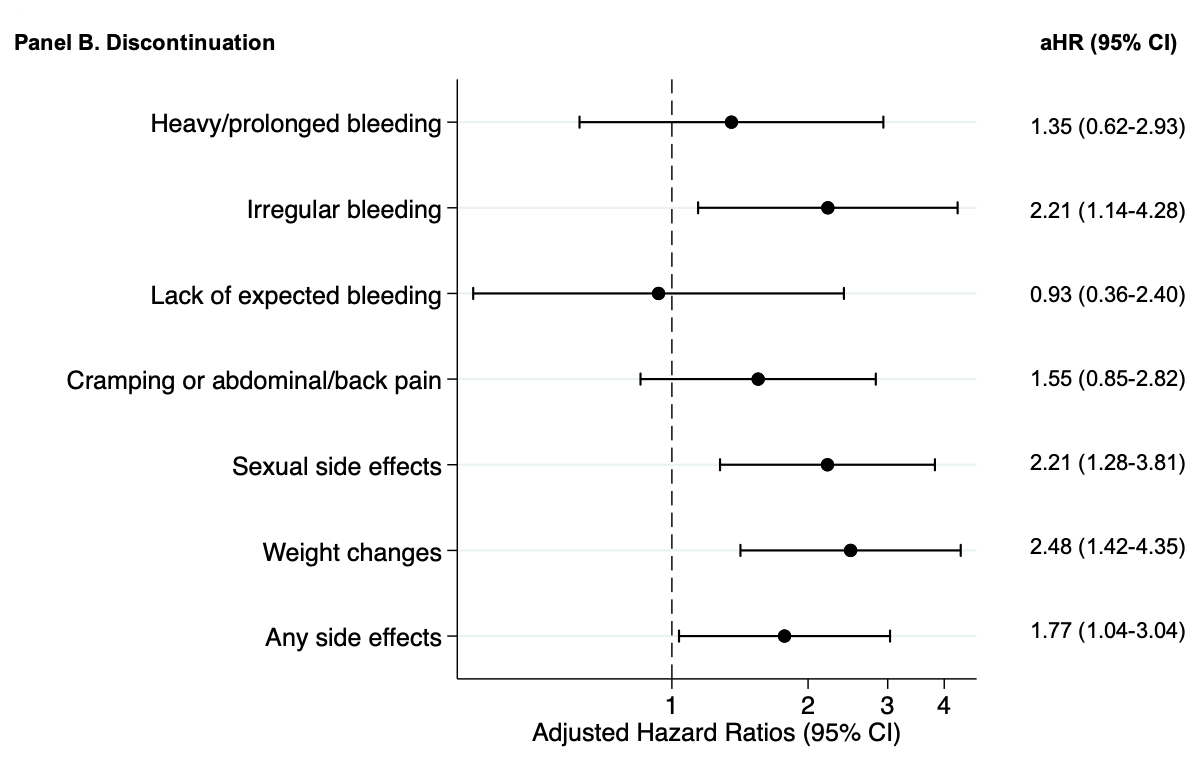


Notes: Cause-specific hazard ratios estimated using Cox proportional hazards models stratified by enrollment facility. All models are adjusted for the following covariates assessed at study enrollment: marital status, contraceptive method type, age (in years), years of completed education, FP user type (initiator, continuer, switcher at baseline), and postpartum status (end of pregnancy within 6 months of study enrollment). Multiple imputation was used to address missingness in report of side effects using the most recent observation in the prior 4 weeks (the primary exposure definition). Imputation models result in some missing values due to missingness in baseline covariates. Adjusted models comprise 646-652 participants due to missing values in covariates included in the imputation model.
