# Supplementary material for: Contributions of side effects to contraceptive discontinuation and method switch among Kenyan women: a prospective cohort study
Source: BJOG. 2022 Jan 18;129(6):926–37. doi: 10.1111/1471-0528.17032 (PMC9035040; doi:10.1111/1471-0528.17032)
Supplement: Supplementary file 5 — Figure S5. Sensitivity analysis: all missing adverse effects exposure values set to ‘unexposed’. [file BJO-129-926-s014.docx]

**S5 Fig: Sensitivity Analysis: All missing side effects exposure values set to “unexposed”**

**Panel A. Method switch**

**Panel B. Discontinuation**

Notes: As in primary models, cause-specific hazard ratios estimated using Cox proportional hazards models stratified by enrollment facility. All models are adjusted for the following baseline covariates: marital status, contraceptive method type, age (in years), years of completed education, FP user type (initiator, continuer, switcher at baseline), and postpartum status (end of pregnancy within 6 months of study enrollment). Specific side effects exposure variables are binary variables coded as 0 for unexposed (no side effect reported) and 1 for exposed (side effect reported). In this sensitivity analysis, all missing side effects were coded as 0.
