# Supplementary material for: Contributions of side effects to contraceptive discontinuation and method switch among Kenyan women: a prospective cohort study
Source: BJOG. 2022 Jan 18;129(6):926–37. doi: 10.1111/1471-0528.17032 (PMC9035040; doi:10.1111/1471-0528.17032)
Supplement: Supplementary file 6 — Figure S6. Effect modification of adverse effects. [file BJO-129-926-s007.docx]

**S5 Fig. Effect modification of side effects**

Panel A. Youth as an effect modifier of switch

Panel B. Strength of desire to avoid pregnancy as an effect modifier of discontinuation

Notes: Cause-specific Cox proportional hazards models include an indictor indicating report of the side effect, an indicator for the effect modifiers (youth in Panel A, strength of desire to avoid pregnancy [DAP] in Panel B), and an interaction term. Desire to avoid pregnancy was defined as strong if women reported that becoming pregnant in the near future would be a "big problem," or weak if a "little problem" or "no problem." All models are additionally adjusted for the following baseline covariates: marital status, FP method at enrollment, years of completed education, FP user type (initiator, continuer, switcher at baseline), and postpartum status at enrollment (defined as end of last pregnancy within 6 year of study enrollment). In Panel B, the analysis excludes women with missing fertility preferences or who expressed that they were unsure if they wanted future children/unsure about the timing of future children, retaining only women with defined intentions for spacing (desire to wait at least 1 year until next pregnancy) or limiting (desire no future children). The model also includes age (in years) as an *a priori* adjustment variable.
