# Supplementary material for: Contributions of side effects to contraceptive discontinuation and method switch among Kenyan women: a prospective cohort study
Source: BJOG. 2022 Jan 18;129(6):926–37. doi: 10.1111/1471-0528.17032 (PMC9035040; doi:10.1111/1471-0528.17032)
Supplement: Supplementary file 7 — Table S1. Adverse effects symptom ascertainment. [file BJO-129-926-s021.docx]

**S1 Table. Side effects symptom ascertainment**

| Side effects symptom category | Relevant question text |
| --- | --- |
| Any side effects | “In the past week, have you had any side effects or problems using ([CURRENT METHOD] if current user or “family planning” if reported stopping FP use in past week)?”  *Note: Participants who responded yes or not sure to this question were asked about specific symptoms using the questions below.* |
| Heavy/prolonged bleeding | “Did you get your monthly periods in the past week?” If YES:  “Were your monthly periods heavier than usual in the past week?” |
| Irregular bleeding | “Did you get your monthly periods in the past week?” If YES:  “Have you had more irregular bleeding or spotting than usual the past week?” |
| Lack of expected bleeding | “Did you get your monthly periods in the past week?” If NO:  Were you expecting your monthly periods in the past week? |
| Abdominal pain, backpain, cramping or painful menses | Have you had more cramping than usual in the past week? |
| Weight changes | Have you had weight changes in the past month? |
| Sexual side effects | Have you had problems with sexual pleasure or desire in the past week?  OR  Have you had pain during intercourse in the past week? |
| Other side effects (hand coded free text) | “Have you had any other side effects or problems using family planning in the past week?” If YES:  “What other side effects or problems have you had in the past week?” [Free text response] |
