# Supplementary material for: Contributions of side effects to contraceptive discontinuation and method switch among Kenyan women: a prospective cohort study
Source: BJOG. 2022 Jan 18;129(6):926–37. doi: 10.1111/1471-0528.17032 (PMC9035040; doi:10.1111/1471-0528.17032)
Supplement: Supplementary file 8 — Table S2. Percent of weeks prior to method switch or discontinuation in which participants reported adverse effects. [file BJO-129-926-s025.docx]

**S2 Table. Percent of weeks prior to method switch or discontinuation in which participants reported side effects**

| Percent of weeks participant reported side effect: | **Any side effects or method problems** | **Heavy/prolonged bleeding** | **Irregular bleeding** | **Lack of expected bleeding** | **Cramps/ abdominal/ back pain** | **Sexual side effects: any** | **Sexual side effects: libido/ pleasure problems** | **Sexual side effects: painful intercourse** | **Weight changes*** |
| --- | --- | --- | --- | --- | --- | --- | --- | --- | --- |
|  | n (%) | n (%) | n (%) | n (%) | n (%) | n (%) |  |  | n (%) |
| 0% (never) | 197 (29) | 442 (65) | 419 (61) | 429 (63) | 345 (50) | 337 (49) | 389 (57) | 411 (60) | 344 (51) |
| >0-25% | 235 (34) | 161 (24) | 171 (25) | 191 (29) | 198 (29) | 190 (28) | 169 (25) | 170 (25) | 194 (29) |
| >25-50% | 100 (15) | 47 (7) | 53 (8) | 38 (6) | 81 (12) | 73 (11) | 58 (9) | 53 (8) | 71 (10) |
| >50-75% | 51 (7) | 17 (2) | 19 (3) | 10 (1) | 19 (3) | 29 (4) | 27 (4) | 21 (3) | 21 (3) |
| >75% | 101 (15) | 15 (2) | 21 (3) | 17 (2) | 41 (6) | 54 (8) | 39 (6) | 4 (29) | 49 (7) |
| No. participants | 684 | 682 | 683 | 685 | 684 | 683 | 682 | 684 | 679 |
| * All side effects were reported as occurring in the past week with the exception of weight changes, which were reported as having occurred in the past month. Notes: Row categories are defined as the percent of weeks an individual participant reported experiencing the side effects (calculated out of weeks with completed surveys). Column percentages represent the percent of women in each category (never reported the side effect to "reported the side effect in >75% of weeks). Participants were censored at the week of all-method discontinuation or the week prior to method switch. As such, side effects reflect those experienced using the initial method type used at study enrollment. Participants who switched methods in the first week of follow-up are excluded from these descriptive summaries, as they were not asked about experience of side effects with their initial method. | | | | | | | | | |
