# Supplementary material for: Contributions of side effects to contraceptive discontinuation and method switch among Kenyan women: a prospective cohort study
Source: BJOG. 2022 Jan 18;129(6):926–37. doi: 10.1111/1471-0528.17032 (PMC9035040; doi:10.1111/1471-0528.17032)
Supplement: Supplementary file 9 — Table S3. Percent of weeks prior to method switch or discontinuation in which participants reported sexual adverse effects, by symptom type. [file BJO-129-926-s017.docx]

**S3 Table. Percent of weeks prior to method switch or discontinuation in which participants reported sexual side effects, by symptom type**

| **Panel A. Any sexual side effects** | | | | |
| --- | --- | --- | --- | --- |
| Percent of weeks participant reported side effect: | **Implant** | **Cu-IUD** | **Injectable** | **OCP** |
|  | n (%) | n (%) | n (%) | n (%) |
| 0% (never) | 145 (47) | 24 (49) | 148 (51) | 20 (59) |
| >0-25% | 97 (32) | 15 (31) | 71 (24) | 7 (21) |
| >25-50% | 32 (10) | 5 (10) | 33 (11) | 3 (9) |
| >50-75% | 10 (3) | 3 (6) | 15 (5) | 1 (3) |
| >75% | 23 (7) | 2 (4) | 27 (9) | 3 (9) |
| No. participants | 307 | 49 | 283 | 34 |
| **Panel B. Problems with libido/sexual pleasure** | | | | |
| Percent of weeks participant reported side effect: | **Implant** | **Cu-IUD** | **Injectable** | **OCP** |
|  | n (%) | n (%) | n (%) | n (%) |
| 0% (never) | 175 (57) | 26 (53) | 168 (57) | 20 (59) |
| >0-25% | 82 (37) | 15 (31) | 65 (22) | 7 (21) |
| >25-50% | 24 (8) | 4 (8) | 26 (9) | 4 (12) |
| >50-75% | 11 (4) | 3 (6) | 13 (4) | 0 (0) |
| >75% | 14 (5) | 1 (2) | 21 (7) | 3 (9) |
| No. participants | 306 | 49 | 293 | 34 |
| **Panel C. Painful intercourse** | |  |  |  |
| Percent of weeks participant reported side effect: | **Implant** | **Cu-IUD** | **Injectable** | **OCP** |
|  | n (%) | n (%) | n (%) | n (%) |
| 0% (never) | 186 (60) | 27 (55) | 171 (58) | 27 (79) |
| >0-25% | 78 (25) | 14 (28) | 74 (25) | 4 (12) |
| >25-50% | 24 (8) | 5 (10) | 23 (8) | 1 (3) |
| >50-75% | 8 (3) | 2 (40) | 10 (3) | 1 (3) |
| >75% | 12 (4) | 1 (2) | 15 (5) | 1 (3) |
| No. participants | 308 | 49 | 293 | 34 |

Notes: Row categories are defined as the percent of weeks an individual participant reported experiencing the side effects (calculated out of weeks with completed surveys). Column percentages represent the percent of women in each category (never reported the side effect to "reported the side effect in >75% of weeks). Participants were censored at the week of all-method discontinuation or the week prior to method switch. As such, side effects reflect those experienced using the initial method type used at study enrollment. Participants who switched methods in the first week of follow-up are excluded from these descriptive summaries, as they were not asked about experience of side effects with their initial method.
