# Supplementary material for: Contributions of side effects to contraceptive discontinuation and method switch among Kenyan women: a prospective cohort study
Source: BJOG. 2022 Jan 18;129(6):926–37. doi: 10.1111/1471-0528.17032 (PMC9035040; doi:10.1111/1471-0528.17032)
Supplement: Supplementary file 10 — Table S4. Contraceptive use dynamics over 24 weeks. [file BJO-129-926-s018.docx]

| **S4 Table. Contraceptive use dynamics over 24 weeks** |  |  |
| --- | --- | --- |
|  |  | **Overall** |
|  | **n** | **n (%) or IR (95% CI)** |
| **Panel A. Method switch** |  |  |
| Incidence of method switch (per 100 woman-years)* | 825 | 61.80 (52.85-72.26) |
| Incidence of method switch by method type(per 100 woman-years)* |  |  |
| Implant | 825 | 52.1 (40.4-67.1) |
| IUD |  | 63.9 (26.4-112.5) |
| Injectable |  | 64.0 (50.6-81.0) |
| OCP |  | 130.5 (80.0-213.1) |
| *Among switchers:* |  |  |
| Switched from LARC to another LARC | 157 | 9 (6) |
| Switched from LARC to non-LARC |  | 63 (40) |
| Switched from non-LARC to LARC |  | 59 (38) |
| Switched from non-LARC to another non-LARC |  | 26 (17) |
| *Stated reason for switch* |  |  |
| Side effects | 157 | 47 (30) |
| More convenient method |  | 10 (6) |
| More effective method |  | 16 (10) |
| Other reason |  | 5 (3) |
| Missing/refused |  | 79 (50) |
| **Panel B. Contraceptive discontinuation** |  |  |
| Incidence of discontinuation (per 100 woman-years)* | 825 | 38.58 (31.65-46.02) |
| Incidence of discontinuation by method type (per 100 woman-years)* | |  |
| Implant | 825 | 28.6 (20.4-40.3) |
| IUD |  | 42.6 (21.3-85.2) |
| Injectable |  | 41.7 (31.2-55.9) |
| OCP |  | 97.9 (55.6-172.4) |
| *Stated reason for discontinuation* |  |  |
| Pregnancy desire | 98 | 13 (13) |
| Side effects |  | 9 (9) |
| Forgot |  | 7 (7) |
| Partner away |  | 3 (3) |
| Other reason |  | 6 (6) |
| Missing/refused |  | 60 (61) |
| Notes: Analytic sample is restricted to women who did not report desiring their next pregnancy within 1 year and who were using implants, injectables, IUD or OCP at baseline; LARC = long-acting reversible contraceptive, which in our sample comprises IUD and implants. *Case-specific incidence rates (IR) are completely unadjusted; parentheses indicate 95% CI. | | |
