# Supplementary material for: Contributions of side effects to contraceptive discontinuation and method switch among Kenyan women: a prospective cohort study
Source: BJOG. 2022 Jan 18;129(6):926–37. doi: 10.1111/1471-0528.17032 (PMC9035040; doi:10.1111/1471-0528.17032)
Supplement: Supplementary file 11 — Table S5. Discontinuation and method switch by baseline characteristics. [file BJO-129-926-s001.docx]

**S5 Table. Discontinuation and method switch by baseline characteristics**

|  | **Discontinuation** | | **Method Switch** | |
| --- | --- | --- | --- | --- |
|  | HR  (95% CI) | aHR  (95% CI) | HR  (95% CI) | aHR  (95% CI) |
| *Method type* |  |  |  |  |
| Implant | *Ref.* | *Ref.* | *Ref.* | *Ref.* |
| IUD | 1.42  (0.65-3.10) | 1.64  (0.72-3.74) | 1.13  (0.60-2.12) | 1.13  (0.59-2.16) |
| Injectable | 1.42  (0.87-2.32) | 1.43  (0.84-2.43) | 1.18  (0.80-1.75) | 1.14  (0.75-1.71) |
| OCP | 2.46  (1.30-4.65) | 2.93  (1.51-5.67) | 2.12  (1.18-3.81) | 1.84  (0.97-3.50) |
| *Desire for pregnancy in the future^a^* |  |  | ` |  |
| None | *Ref.* | *Ref.* | *Ref.* | *Ref.* |
| Not sure | 0.39  (0.13-1.14) | 0.46  (0.15-1.34) | 0.78  (0.40-1.53) | 0.79  (0.40-1.54) |
| Yes, but unsure when | 0.95  (0.47-1.91) | 0.81  (0.34-1.92) | 0.85  (0.46-1.56) | 0.68  (0.35-1.32) |
| Yes, in 1-2 years | 1.45  (0.77-2.75) | 1.40  (0.66-2.94) | 1.19  (0.66-2.15) | 1.01  (0.55-1.86) |
| Yes, in >2 years | 0.70  (0.43-1.14) | 0.79  (0.42-1.47) | 1.04  (0.70-1.56) | 0.86  (0.54-1.36) |
| *FP user type at baseline* |  |  |  |  |
| Initiator | *Ref.* | *Ref.* | *Ref.* | *Ref.* |
| Switcher | 1.47  (0.67-3.21) | 1.18  (0.50-2.78) | 0.74  (0.42-1.32) | 0.72  (0.40-1.30) |
| Continuer | 1.34  (0.79-2.25) | 1.25  (0.71-2.21) | 0.76  (0.53, 1.09) | 0.76  (0.52-1.13) |
| Received all 3 components of method information index (MII)^a^ *(ref: <3)* | 1.12  (0.69-1.80) | 1.34  (0.80-2.27) | 1.32  (0.91-1.92) | 1.34  (0.88-2.05) |
| Cu-IUD = intrauterine device, OCP = daily oral contraceptive pills | | | | |
| ^a^ Women who want to become pregnant within the next 1 year were excluded from the analysis in order to improve the sensitivity of our analyses of side effects to focus on method-related and unplanned contraceptive discontinuation | | | | |
| ^b^The MII is coded as a binary variable equal to 1 if women reported received all 3 of the following counseling components during the FP visit on the date of study enrollment (or at her most recent visit, if she did not receive FP services on the date of enrollment): information on other methods, side effects of selected method, and what to do if side effects occur; the variables is coded as 0 if <3 counseling items were reported. | | | | |
| Notes: adjusted cause-specific Cox proportional hazards models include the following covariates: method type at enrollment, marital status, age (years), completed education (years), FP user type, and postpartum status at enrollment (end of last pregnancy <6 months ago). All models (unadjusted and adjusted) stratified by enrollment facility. | | | | |
