# Supplementary material for: Contributions of side effects to contraceptive discontinuation and method switch among Kenyan women: a prospective cohort study
Source: BJOG. 2022 Jan 18;129(6):926–37. doi: 10.1111/1471-0528.17032 (PMC9035040; doi:10.1111/1471-0528.17032)
Supplement: Supplementary file 12 — Table S6. Incidence of modern‐method discontinuation and switch. [file BJO-129-926-s005.docx]

**S6 Table. Incidence of modern-method discontinuation and switch**

|  | **Modern-Method Discontinuation** | | |  | **Method Switch** | | |
| --- | --- | --- | --- | --- | --- | --- | --- |
|  | Number of events | Person-time observed (years) | Incidence rate* (95% CI) |  | Number of events | Person-time observed (years) | Incidence rate* (95% CI) |
| Overall | 98 | 254.3 | 38.5 (31.6, 47.0) |  | 156 | 254.3 | 61.3 (52.4, 71.8) |
| *Method used at enrollment:* |  |  |  |  |  |  |  |
| Implant | 33 | 115.5 | 28.6 (20.3, 40.2) |  | 59 | 115.5 | 51.1 (39.6, 65.9) |
| IUD | 8 | 18.8 | 42.6 (21.3, 85.2) |  | 12 | 18.8 | 63.9 (36.3, 112.5) |
| Injectable | 45 | 107.8 | 41.8 (31.2, 56.0) |  | 69 | 107.8 | 64.1 (50.6, 81.1) |
| OCP | 12 | 12.3 | 97.9 (55.6, 172.4) |  | 16 | 12.3 | 130.5 (80.0, 213.1) |
| * per 100 woman-years |  |  |  |  |  |  |  |
