# Supplementary material for: Contributions of side effects to contraceptive discontinuation and method switch among Kenyan women: a prospective cohort study
Source: BJOG. 2022 Jan 18;129(6):926–37. doi: 10.1111/1471-0528.17032 (PMC9035040; doi:10.1111/1471-0528.17032)
Supplement: Supplementary file 13 — Table S7. Effect modification of adverse effects on switch and discontinuation. [file BJO-129-926-s010.docx]

**S7 Table. Effect modification of side effects on switch and discontinuation**

|  | **Discontinuation** | |  | **Switch** | |  |
| --- | --- | --- | --- | --- | --- | --- |
|  | **aHR** | **p-value** |  | **aHR** | **p-value** |  |
| **Panel A. Desire to avoid pregnancy** |  |  |  |  |  |  |
| ***Heavy bleeding*** |  |  |  |  |  |  |
| Strong desire | 0.76 | 0.46 |  | 1.00 | 0.99 |  |
| Heavy bleeding | 1.94 | 0.28 |  | 0.95 | 0.96 |  |
| Heavy bleeding*Strong desire | 0.84 | 0.83 |  | 2.32 | 0.43 |  |
| ***Irregular bleeding*** |  |  |  |  |  |  |
| Strong desire | 1.05 | 0.91 |  | 1.12 | 0.79 |  |
| Irregular bleeding | 4.83 | 0.002 |  | 2.51 | 0.14 |  |
| Irregular bleeding*Strong desire | 0.28 | 0.12 |  | 0.90 | 0.89 |  |
| ***Lack of expected bleeding*** |  |  |  |  |  |  |
| Strong desire | 0.64 | 0.21 |  | 0.94 | 0.88 |  |
| Lack of expected bleeding | 0.41 | 0.44 |  | 2.57 | 0.15 |  |
| Lack of expected bleeding*Strong desire | 5.00 | 0.22 |  | 1.05 | 0.95 |  |
| ***Cramping or abdominal/back pain*** |  |  |  |  |  |  |
| Strong desire | 0.92 | 0.84 |  | 0.82 | 0.65 |  |
| Cramping or abdominal/back pain | 3.78 | 0.003 |  | 0.64 | 0.54 |  |
| Cramping or abdominal/back pain*Strong desire | 0.50 | 0.27 |  | 2.84 | 0.22 |  |
| ***Sexual side effects*** |  |  |  |  |  |  |
| Strong desire | 0.95 | 0.90 |  | 0.89 | 0.79 |  |
| Sexual side effects | 3.82 | 0.003 |  | 1.70 | 0.32 |  |
| Sexual side effects*Strong desire | 0.34 | 0.10 |  | 1.47 | 0.55 |  |
| ***Weight changes*** |  |  |  |  |  |  |
| Strong desire | 0.79 | 0.56 |  | 1.00 | 0.99 |  |
| Weight changes | 2.81 | 0.04 |  | 1.83 | 0.30 |  |
| Weight changes*Strong desire | 0.79 | 0.72 |  | 0.93 | 0.92 |  |
| ***Any side effects*** |  |  |  |  |  |  |
| Strong desire | 0.75 | 0.52 |  | 0.91 | 0.83 |  |
| Any side effects | 2.09 | 0.11 |  | 1.40 | 0.50 |  |
| Any side effects*Strong desire | 0.80 | 0.71 |  | 1.34 | 0.62 |  |
| **Panel B. Young adulthood (<25 years old)** |  |  |  |  |  |  |
| ***Heavy bleeding*** |  |  |  |  |  |  |
| Young adult | 0.75 | 0.38 |  | 1.76 | 0.04 |  |
| Heavy bleeding | 1.03 | 0.96 |  | 3.41 | 0.003 |  |
| Heavy bleeding*young adult | 2.71 | 0.23 |  | 0.10 | 0.04 |  |
| ***Irregular bleeding*** |  |  |  |  |  |  |
| Young adult | 0.83 | 0.57 |  | 1.63 | 0.08 |  |
| Irregular bleeding | 2.16 | 0.10 |  | 2.28 | 0.06 |  |
| Irregular bleeding*young adult | 1.39 | 0.64 |  | 0.27 | 0.15 |  |
| ***Lack of expected bleeding*** |  |  |  |  |  |  |
| Young adult | 0.84 | 0.56 |  | 1.61 | 0.08 |  |
| Lack of expected bleeding | 0.80 | 0.71 |  | 3.17 | 0.003 |  |
| Lack of expected bleeding*young adult | 2.27 | 0.41 |  | 0.47 | 0.27 |  |
| ***Cramping or abdominal/back pain*** |  |  |  |  |  |  |
| Young adult | 0.63 | 0.20 |  | 2.18 | 0.007 |  |
| Cramping, abdominal/back pain | 1.18 | 0.69 |  | 2.91 | 0.003 |  |
| Cramping or abdominal/back pain*young adult | 3.27 | 0.07 |  | 0.00 | -- |  |
| ***Sexual side effects*** |  |  |  |  |  |  |
| Young adult | 0.73 | 0.38 |  | 2.26 | 0.006 |  |
| Sexual side effects | 2.10 | 0.04 |  | 3.53 | <0.001 |  |
| Sexual side effects*young adult | 1.58 | 0.46 |  | 0.10 | 0.006 |  |
| ***Weight changes*** |  |  |  |  |  |  |
| Young adult | 0.80 | 0.53 |  | 1.75 | 0.05 |  |
| Weight changes | 2.29 | 0.02 |  | 1.87 | 0.12 |  |
| Weight changes*young adult | 1.51 | 0.51 |  | 0.34 | 0.16 |  |
| ***Any side effects*** |  |  |  |  |  |  |
| Young adult | 0.71 | 0.39 |  | 2.39 | 0.005 |  |
| Any side effects | 1.57 | 0.19 |  | 2.71 | 0.003 |  |
| Any side effects*young adult | 1.60 | 0.43 |  | 0.22 | 0.010 |  |
| Notes: Cause-specific hazard ratios of discontinuation and method switch estimated using separate Cox proportional hazards models stratified by enrollment facility and adjusted for the following covariates: marital status, contraceptive method at enrollment, years of completed education, and FP user type (initiator, continuer, switcher at baseline) and early postpartum status (end of most recent pregnancy <6 months ago). Models except in which young adulthood is the exposure of interest adjust for age in years. Models of strength of desire to avoid pregnancy exclude participants who reported uncertainty in fertility intentions at baseline (unsure if want future pregnancy, unsure of ideal timing of next pregnancy, or not sure how big of a problem a near-term pregnancy would be). DAP is defined as "strong" if the participant reported that becoming pregnant in the near future would be a "big problem" or weak if a "small problem" or "no problem." * symbol denotes coefficient on the interaction term for side effect and potential effect modifier. | | | | | |  |
|  |  |  |  |  |  |  |
|  |  |  |  |  |  |  |
|  |  |  |  |  |  |  |
|  |  |  |  |  |  |  |
|  |  |  |  |  |  |  |
|  |  |  |  |  |  |  |
|  |  |  |  |  |  |  |
|  |  |  |  |  |  |  |
|  |  |  |  |  |  |  |
|  |  |  |  |  |  |  |
|  |  |  |  |  |  |  |

Notes: Cause-specific hazard ratios of discontinuation and method switch estimated using separate Cox proportional hazards models stratified by enrollment facility, with switch or discontinuation defined as the competing risk. All models are stratified by enrollment facility and adjusted for the following covariates: marital status, contraceptive method at enrollment, years of completed education, and FP user type (initiator, continuer, switcher at baseline) and early postpartum status (end of most recent pregnancy <6 months ago). Models except in which young adulthood is the exposure of interest adjust for age in years. Models of strength of desire to avoid pregnancy (DAP) exclude participants who reported uncertainty in fertility intentions at baseline (unsure if want future pregnancy, unsure of ideal timing of next pregnancy, or not sure how big of a problem a near-term pregnancy would be). DAP is defined as "strong" if the participant reported that becoming pregnant in the near future would be a "big problem" or weak if a "small problem" or "no problem." * symbol denotes coefficient on the interaction term for side effect and potential effect modifier.
