# Supplementary material for: Contributions of side effects to contraceptive discontinuation and method switch among Kenyan women: a prospective cohort study
Source: BJOG. 2022 Jan 18;129(6):926–37. doi: 10.1111/1471-0528.17032 (PMC9035040; doi:10.1111/1471-0528.17032)
Supplement: Supplementary file 14 — Table S8. Associations between level method satisfaction and switch and discontinuation. [file BJO-129-926-s024.docx]

**S8 Table. Associations between level method satisfaction and switch and discontinuation**

| **Panel A. Method Switch** | |  |
| --- | --- | --- |
|  | **aHR (95% CI)** | **p-value** |
| *Method satisfaction* |  |  |
| Very satisfied | *Ref.* |  |
| Satisfied | 0.76 (0.41-1.42) | 0.394 |
| Neutral | 1.82 (0.89-3.72) | 0.103 |
| Dissatisfied | 2.19 (0.96-5.01) | 0.063 |
| Very dissatisfied | 1.29 (0.16-10.24) | 0.811 |
| Any side effects | 1.21 (0.73-2.00) | 0.468 |
|  |  |  |
| **Panel B. Discontinuation** | |  |
|  | **Model 2** | |
|  | **aHR (95% CI)** | **p-value** |
| *Method satisfaction* |  |  |
| Very satisfied | *Ref.* |  |
| Satisfied | 0.86 (0.43-1.71) | 0.674 |
| Neutral | 1.60 (0.72-3.59) | 0.251 |
| Dissatisfied | 0.88 (0.25-3.11) | 0.845 |
| Very dissatisfied | 4.54 (1.30-15.88) | 0.018 |
| Any side effects | 1.60 (0.89-2.87) | 0.116 |
| Notes: In addition to the categorical method satisfaction variable and an indicator of any side effects experienced, Cox PH models additionally include *a priori* adjustment variables: married (legal or presumed), method type, age (in years), years of education, FP user type at enrollment (initiator, switcher or continuer), and <6 months since end of last pregnancy. Participants switching methods or discontinuing all modern contraception in week 1 of follow-up are excluded, as satisfaction with the original method was not ascertained. | | |
