# Supplementary material for: Contributions of side effects to contraceptive discontinuation and method switch among Kenyan women: a prospective cohort study
Source: BJOG. 2022 Jan 18;129(6):926–37. doi: 10.1111/1471-0528.17032 (PMC9035040; doi:10.1111/1471-0528.17032)
Supplement: Supplementary file 15 — Appendix S1. Details of study design, procedures and variable ascertainment. [file BJO-129-926-s002.docx]

**Contributions of side effects to contraceptive discontinuation and method switch among Kenyan women: a prospective cohort study**

**Supplementary Material**

**DETAILS OF STUDY DESIGN, PROCEDURES, AND VARIABLE ASCERTAINMENT**

**Study enrollment**

After screening for eligibility, eligible participants completed a written informed consent. Women who completed informed consent were defined as enrolled in the study based on successful completion of two SMS-based survey items on their own mobile phones: 1) an opt-in screening question, which asked the participant to select 1 to continue with the survey, and 2) by selecting her preferred language (English, Swahili, Luo, or Kisii). Completion of these two questions was required to trigger the automated weekly SMS follow-up surveying. As a result, participants who failed to complete these questions on their own mobile phone (either intentionally or due to survey timeout, which was noted by study staff in instances of mobile network issues) did not receive follow-up messaging and so were not considered enrolled in the study despite having completed informed consent.

**Definition of method switch and discontinuation**

To reduce measurement error in ascertainment of method switching, method switch was defined based on the following requirements: 1) apparent switches (weeks in which a method type that differed from that used at enrollment was reported) were recoded as “0” (non-switches) if the participant reported that she did not switch methods in the past week and did not report switching methods at any time in the following 4 weeks; and 2) the participant was required to report use of the new method type for at least four consecutive weeks. The purpose of these requirements was to limit misclassification due to participant entry error, which is possible given the remote, self-questionnaire mode of data collection.

Contraceptive discontinuation is defined as a period of 2 or more consecutive weeks in which the participant reports use of either no contraceptive method or a traditional contraceptive method. It is therefore a measure of discontinuation of all modern methods of contraception. To operationalize this definition, we made several assumptions about method use reported over weekly surveys: 1) weekly surveys in which the participant reported using contraceptive but for which the method type is missing or refused (comprising 0.20% [27/13,229] weekly observations using the LOCF/NOCB singly imputed dataset) were considered to represent continued use of the initial method; such instances were not considered method switches or discontinuation if occurring in consecutive weeks; 2) fertility-awareness based (FAB) methods (included lactational amenorrhea) were reporting using the common terms “counting days” or “breastfeeding method (before return of menses after childbirth)”; since use of a modern method was required for study enrollment, we assume that women reporting “counting days” as their contraceptive method during follow-up (n=20/13,229, 0.15%) were using a modern FAB method.

**Ascertainment of contraceptive side effects**

Each week, study participants who reported current method use or discontinuation in the past 7 days were asked if they had experienced any side effects or problems using that method in the past week using the question text, “In the past week, have you had any side effects or problems using [*method type*]?” We defined overall experience of any side effects or method problems in the past week and month as a binary variable equal to 1 if the participant reported “yes,” 0 if the participant reported “no”, and missing in the case of “not sure,” declined, or incomplete responses.

We ascertained experience of specific side effects using a series of follow-up questions tailored to contraceptive method type. Relevant questions used to ascertain specific side effects were asked only of women reporting “yes” or “not sure” to the question about experiencing any side effects or problems with their method in the past week (S1 Table). These women were also asked a final question about other symptoms experienced (“Have you had any other side effects or problems using family planning in the past week?”), which allowed women to write in symptoms in a free text response. These responses were them translated as needed and categorized within the symptom categories where relevant by the U.S.- and Kenya-based study coordinators. In some cases, frequent free text responses were incorporated into a larger category: for example, while there was no specific question for abdominal or back pain, free-text responses about these symptoms were grouped into the “cramping” symptom category.

Specific side effects were each coded as a binary variable equal to 1 if the woman reported experiencing the symptom, or 0 if either 1) the woman reported no experiencing any side effects or method problems in the past week, or 2) she reported experiencing side effects but not that specific symptoms. Women were coded as missing in the case of “not sure”, missing, or declined responses to the specific symptom, or if her response to the overall question about any side effects was missing or refused.

**Ascertainment of sociodemographic and reproductive health characteristics**

Sociodemographic characteristics and reproductive and contraceptive history were assessed at the time of study enrollment. Baseline fertility intentions were defined categorically as desiring no future children, unsure about future children, desiring future children but unsure of preferred timing, or desiring a pregnancy in 1-2 years or in >2 years. Women stating a desired pregnancy within 1 year were excluded from analyses, in order to focus on method-related rather than planned discontinuation.
