# Supplementary material for: Contributions of side effects to contraceptive discontinuation and method switch among Kenyan women: a prospective cohort study
Source: BJOG. 2022 Jan 18;129(6):926–37. doi: 10.1111/1471-0528.17032 (PMC9035040; doi:10.1111/1471-0528.17032)
Supplement: Supplementary file 25 — Supplementary Material [file BJO-129-926-s009.docx]

**ICMJE DISCLOSURE FORM**

**Date:** October 24, 2021

**Your Name:** Claire Watt Rothschild

**Manuscript Title:** Contributions of side effects to contraceptive discontinuation and method switch among Kenyan women: a prospective cohort study

**Manuscript number (if known):** BJOG-21-0348

**In the interest of transparency, we ask you to disclose all relationships/activities/interests listed below that are**

**related to the content of your manuscript. “Related” means any relation with for-profit or not-for-profit third**

**parties whose interests may be affected by the content of the manuscript. Disclosure represents a commitment**

**to transparency and does not necessarily indicate a bias. If you are in doubt about whether to list a relationship/activity/interest, it is preferable that you do so.**

**The following questions apply to the** **author’s relationships/activities/interests as they relate to the current**

**manuscript only.**

**The author’s relationships/activities/interests should be defined broadly. For example, if your manuscript pertains**

**to the epidemiology of hypertension, you should declare all relationships with manufacturers of antihypertensive medication, even if that medication is not mentioned in the manuscript.**

**In item #1 below, report all support for the work reported in this manuscript without time limit. For all other items,**

**the time frame for disclosure is the past 36 months.**

|  |  | **Name all entities with whom you have this relationship or indicate none (add rows as needed)** | | **Specifications/Comments**  **(e.g., if payments were made to you or to your institution)** |
| --- | --- | --- | --- | --- |
| **Time frame: Since the initial planning of the work** | | | | |
| 1 | All support for the present manuscript (e.g., funding, provision of study materials, medical writing, article processing charges, etc.)  **No time limit for this item.** | ____ None | |  |
|  |  | Gates Grand Challenges Explorations (OPP1172004) | | Grant made to Alison L. Drake (PI) |
|  |  | Eunice Kennedy Shriver National Institute of Child Health & Human Development of the National Institutes of Health [NICHD] (F31HD097841 | | Pre-doctoral fellowship for Dr. Claire Rothschild, which supported the development of this manuscript |
|  |  | Bill & Melinda Gates Foundation | | Funding for Open Publishing |
|  |  | NICHD (P2C HD042828) | | Research infrastructure grant to the Center for Studies in Demography & Ecology at the University of Washington |
|  |  | NICHD (T32 HD101442-01) | | Training grant to the Center for Studies in Demography & Ecology at the University of Washington |
|  |  |  | |  |
|  |  |  | |  |
| **Time frame: past 36 months** | | | | |
| 2 | Grants or contracts from any entity (if not indicated in item #1 above). | __X__ None |  | |
|  |  |  |  | |
|  |  |  |  | |
| 3 | Royalties or licenses | __X__ None |  | |
|  |  |  |  | |
|  |  |  |  | |
| 4 | Consulting fees | ____ None |  | |
|  |  | Bill & Melinda Gates Foundation | Dr. Rothschild served as a subject matter expert consultant with the BMGF’s family planning data team to support the Foundation’s family planning results framework and monitoring strategy to evaluate the Foundation’s investments in family planning. The work was unrelated to the content of this manuscript. | |
|  |  |  |  | |
| 5 | Payment or honoraria for lectures, presentations, speakers bureaus, manuscript writing or educational events | __X__ None |  | |
|  |  |  |  | |
|  |  |  |  | |
| 6 | Payment for expert testimony | __X__ None |  | |
|  |  |  |  | |
|  |  |  |  | |
| 7 | Support for attending meetings and/or travel | __X__ None |  | |
|  |  |  |  | |
|  |  |  |  | |
| 8 | Patents planned, issued or pending | __X__ None |  | |
|  |  |  |  | |
|  |  |  |  | |
| 9 | Participation on a Data  Safety Monitoring Board or Advisory Board | __X__ None |  | |
|  |  |  |  | |
|  |  |  |  | |
| 10 | Leadership or fiduciary role in other board, society, committee or advocacy group, paid or unpaid | ___X_ None |  | |
|  |  |  |  | |
|  |  |  |  | |
| 11 | Stock or stock options | __X__ None |  | |
|  |  |  |  | |
|  |  |  |  | |
| 12 | Receipt of equipment, materials, drugs, medical writing, gifts or other services | __X__ None |  | |
|  |  |  |  | |
|  |  |  |  | |
| 13 | Other financial or non-financial interests | __X__ None |  | |
|  |  |  |  | |
|  |  |  |  | |

**Please place an “X” next to the following statement to indicate your agreement:**

**_X__ I certify that I have answered every question and have not altered the wording of any of the questions on this**

**form.**
